# Association of salivary Cathepsin B in different histological grades among patients presenting with oral squamous cell carcinoma

**DOI:** 10.1186/s12903-022-02052-1

**Published:** 2022-03-08

**Authors:** Alveena Shabbir, Humera Waheed, Shaheen Ahmed, Sabhita Shabir Shaikh, Waqas Ahmed Farooqui

**Affiliations:** 1 Department of Oral Medicine, Dow University of Health Sciences, Karachi, Pakistan; 2 Department of Dow College of Biotechnology, Dow University of Health Sciences, Karachi, Pakistan; 3 Department of Oral and Maxillofacial Surgery, Dow University of Health Sciences, Karachi, Pakistan; 4 Department of NILGID, Dow University of Health Sciences, Karachi, Pakistan; 5 Department of School of Public Health, Dow University of Health Sciences, Karachi, Pakistan

**Keywords:** Oral squamous cell carcinoma, Saliva, ELISA, Cathepsin B, Biomarker, Histological grades

## Abstract

**Background:**

Oral cancer is considered a major public health problem due to its high mortality and morbidity rates. Survival rate of OSCC can be significantly improved by using non-invasive tool such as salivary biomarkers for detection of OSCC which is considered a promising approach. Cathepsin B is a lysosomal cysteine protease, present in abundant quantities in lysosome of cells, tissues and different biological fluids. Increased expression of Cathepsin B was observed in many malignancies including oral cancer. The present study was designed to determine the salivary levels of Cathepsin B in different histological grades of OSCC.

**Method:**

In this study, total no. of 80 research participants were enrolled which were divided into four groups. Each group comprised 20 participants, group 1 comprised 20 patients of OSCC (well differentiated), group 2 comprised 20 patients of OSCC (moderately differentiated), group 3 comprised 20 patients of OSCC (poorly differentiated) and group 4 comprised 20 healthy controls. Saliva sample was collected from all the four study groups and salivary Cathepsin B levels were analyzed by ELISA sandwich technique in duplicate.

**Results:**

Salivary levels of Cathepsin B were significantly increased with *p* value (< 0.001) in patients of OSCC as compared to control group according to both histological grades and tumor size. Highest mean Cathepsin B levels in well differentiated OSCC followed by poorly differentiated OSCC and moderately differentiated OSCC were observed.

**Conclusion:**

Results of the present study suggests that Cathepsin B has a great value as a salivary biomarker for diagnosis and monitoring of OSCC in different histological grades. This will further lead to increase survival rate and improve the prognosis of OSCC.

## Introduction

Oral cancer is a malignant neoplasm which is considered as a major and alarming health problem of global concern due to increasing prevalence and high mortality rate [1]. It is the sixth most common and leading cause of mortality worldwide and ranked as the 2nd most common cancer in Pakistan [2, 3]. Oral squamous cell carcinoma (OSCC) is the most common and frequent type of oral cancer which accounts for approximately 90% of all oral cancers [4, 5]. Annual incidence rate of oral cancer is 300,000 cases and mortality rate is 145,000 cases worldwide approximately [6]. Most common sites of OSCC occurrence are buccal mucosa, tongue and floor of the mouth [7]. The most important and prevalent risk factors for development of OSCC are tobacco, alcohol and smokeless tobacco chewing [8]. The other risk factors include: viral infections HPV16 and HPV18, genetic factors, occupational exposure, ultraviolent radiation and nutritional deficiencies due to low consumption of fruits and vegetables [9]. The main treatment modalities currently available for OSCC are surgical excision, radiotherapy and chemotherapy [10].

In spite of the advancement in treatment regimens, the 5-year survival rate of OSCC is found to be less than 50% [11]. Although examination of oral cavity is easily assessable but most OSCC are still diagnosed in an advanced stages [12]. Currently, gold standard for the diagnosis of OSCC is histopathological examination of a biopsy sample [13]. However, biopsy is an invasive procedure and has various drawbacks such as painful, high cost and fear of cancer spread after performing biopsy which results in late diagnosis of OSCC [14]. Therefore, there is an urgent need to identify new non-invasive methods which will help us in diagnosis and monitoring of OSCC [15]. Salivary biomarkers are considered a promising and ideal tool in this regard for diagnosis and monitoring of OSCC [16]. Using saliva as a diagnostic tool has several advantages like collection of saliva is simple, safe, non-invasive in nature and cost effective. It also has benefits of ease in sampling, handling and processing [17, 18]. Furthermore, due to direct contact between saliva and oral cavity salivary biomarkers are considered a perfect and classic tool for diagnosis and monitoring of OSCC [19]. Several salivary biomarkers in oral squamous cell carcinoma have been identified which can be used as diagnostic and prognostic markers in OSCC [20].

Cathepsin B (CTSB) is a lysosomal cysteine protease and belongs to a family of cysteine cathepsins. It is present in abundant quantities in lysosomes of cells and different biological fluids of body such as saliva and serum [21]. Main physiological function of Cathepsin B is intracellular degradation and turnover of proteins [22]. Cathepsin B plays a major and important role in development of cancer and its progression. It is associated with basement membrane dissolution and extracellular matrix degradation, a process responsible for tumor progression, growth, metastasis and invasion [23]. Its expression in tissues and serum levels was increased in many types of malignancies including OSCC [24]. However, previously none of the studies have reported Cathepsin B levels in saliva in patients of OSCC in different histological grades. Therefore, analyzing Cathepsin B levels in saliva might be beneficial in diagnosis and monitoring of OSCC by using non-invasive technique. This will lead to increase survival rate and further improve the prognosis of the disease.

## Materials and methods

### Study design and setting

An analytical cross-sectional study was conducted during the period of July 2019 to December 2019 after approval from Institutional Review Board (IRB) of Dow University of Health Sciences (IRB-1223/DUHS/Approval/2019/34). The recruitment of study participants for OSCC was carried out at dental OPD of Dow University of Health Sciences. Recruitment of control group participants was carried out from patient’s attendant’s, friends, family and colleagues. Informed consent in written was obtained from each research participants.

### Sample size estimation

The power of the test was calculated to justify the sample size of 20 subjects in each group using PASS version 15 software, based on One-way ANOVA with 95% confidence of interval, 1.30 standard deviation of means, 2.08 standard deviation of Cathepsin B. It was achieved more than 99%.

### Study groups

Total no. of 80 research participants were enrolled in our study which were divided into four groups 20 in each group. Group 1 consists of 20 patients of well differentiated OSCC, group 2 consists of 20 patients of moderately differentiated OSCC, group 3 consists of 20 patients of poorly differentiated OSCC and group 4 consists of 20 healthy controls. Inclusion criteria for OSCC patients includes: patients of OSCC of both genders, aged 18–65, newly diagnosed and biopsy proven were selected. OSCC patients were divided respectively into three groups i.e., well, moderate and poorly differentiated according to Broder’s histopathological grading criteria. Patients with underlying systemic illness such as rheumatoid arthritis, osteoarthritis, pancreatitis, chronic atrophic gastritis, periodontitis, other malignant tumors such as breast, ovary, lung, gastric and nasopharyngeal were excluded. Patients with a history of smoking and smokeless tobacco products were excluded from the control group. In a pre-designed questionnaire demographic data, type, frequency, duration of chewing habits and site of lesion was obtained for each research participant of OSCC.

### Saliva collection method

In a sterilized falcon tube of 15 ml, 2–5 ml of unstimulated whole saliva was collected from each research participant between 9 and 11 am. Participants were asked to refrain from eating, chewing and drinking one hour prior to saliva sample collection. After that, saliva samples were immediately taken to laboratory and centrifuged for 15 min, 4 °C at 8000 rpm. Saliva supernatant was collected, aliquoted into 1.5 ml of eppendorf tubes and stored at − 80 °C until further processing of saliva.

### Sample analysis

Total protein estimation analysis was performed according to Bradford’s method [26]. Salivary levels of Cathepsin B in all the four study groups were determined by Enzyme Linked Immunosorbent Assay (ELISA) sandwich technique according to manufacturer’s instructions available in the kit (Bioassay Technology KIT: 35054Hu). Each sample was analyzed in duplicates for better result outcomes.

### Statistical analysis

Statistical analysis of all the obtained data was performed by SPSS version no 26. For all quantitative variables, mean and standard deviation were calculated and for all categorical variables, percentages and frequencies were calculated. Kruskal–Wallis test was used for comparison of salivary Cathepsin B levels between three study groups of OSCC and control group. For pair wise comparison, between three groups of OSCC and control group Post Hoc (Dunnett’s test) was used. For patient related factors, association between three OSCC groups and control group Chi-square test and Fisher’s exact test were used. For univariate and multivariate analysis, linear regression was used as the outcome variable Cathepsin B follows a continuous scale. Receiver Operating Characteristic (ROC) curve analysis was performed to determine the cut-off values of Cathepsin B for calculating sensitivity and specificity.

## Results

Clinicopathological characteristics of all 80 research participants enrolled in our study are summarized in Tables 1 and 2. Among the total 60 patients of OSCC, male patients comprised 43 (71.66%) and female patients comprised 17 (28.3%) cases. The male to female ratio observed was 2.5:1. With respect to age, the mean age of 80 research participants was 43.75 ± 11.83 with the minimum 19 years of age and maximum 65 years of age were observed. The most common site found in our study among three groups of OSCC was buccal mucosa 31 cases, followed by tongue 10 cases, retromolar area 5 cases, palate and lower buccal sulcus 4 cases each, upper sulcus and lower lip 3 cases each. Furthermore, we observed that usage of tobacco products for greater than 5 years duration caused increased in no. of cases in all three groups of OSCC.

**Table 1:** Clinicopathological characteristics

Category	N = 80 (%)
Study groups	
Healthy controls	20 (25)
Well differentiated OSCC	20 (25)
Moderately differentiated OSCC	20 (25)
Poorly differentiated OSCC	20 (25)
Tumor size	
Healthy controls	20 (25)
T1	5 (6.3)
T2	9 (11.3)
T3	17 (21.3)
T4	29 (36.3)
Age in years (mean ± SD)	43.75 ± 11.83
Gender	
Male	55 (68.8)
Female	25 (31.3)
Oral habits	
No habits	20 (25)
Pan	7 (8.8)
Guthka	9 (11.3)
Betel Nuts	10 (12.5)
Smoking	11 (13.8)
Betel Nuts, Pan	7 (8.8)
Betel Nuts, Guthka	1 (1.3|)
Betel Nuts, Naswar	2 (2.5)
Betel nuts, Pan, Guthka	2 (2.5)
Guthka, Smoking	1 (1.3)
Guthka, Pan	1 (1.3)
Guthka, Naswar, Smoking	1 (1.3)
Naswar, Smoking	1 (1.3)
Frequency	
No habits	20 (25)
1–5/day	16 (20)
6–10/day	12 (15)
11–15/day	20 (25)
16–20/day	12 (15)
Duration	
No habits	20 (25)
< 5 years	5 (6.3)
6–10 years	11 (13.8)
11–15 years	23 (28.7)
16–20 years	12 (15)
Site	
Missing (healthy controls)	20 (25)
Buccal mucosa	31 (38.8)
Tongue	10 (12.5)
Lower buccal sulcus	4 (5)
Lower lip	3 (3.8)
Palate	4 (5)
Retromolar area	5 (6.3)
Upper sulcus	3 (3.8)

**Table 2:** Clinicopathological data of OSCC patients and Healthy controls

Category	Healthy controls N = 20 (%)	Well differentiated OSCC N = 20 (%)	Moderate differentiated OSCC N = 20 (%)	Poorly differentiated OSCC N = 20 (%)	*p* value
Age in years^^^	37.5 (19)	47.5 (16)	48 (20)	42 (19)	0.17^Ò^
Gender					
Male	12 (21.8)	17 (30.9)	14 (25.5)	12 (21.8)	0.27*
Female	8 (32)	3 (12)	6 (24)	8 (32)	
Habits					
No Habits	20 (100)	0 (0)	0 (0)	0 (0)	_
Pan/Naswar	0 (0)	7 (50)	4 (28.6)	3 (21.4)	
Guthka	0 (0)	2 (22.2)	3 (33.3)	4 (44.4)	
Betel Nuts	0 (0)	1 (10)	5 (50)	4 (40)	
Smoking	0 (0)	4 (36.4)	3 (27.3)	4 (36.4)	
≥ Two habits	0 (0)	6 (37.5)	5 (31.3)	5 (31.3)	
Frequency					
1–5/day	0 (0)	4 (25)	4 (25)	8 (50)	0.59**
6–10/day	0 (0)	5 (41.7)	5 (41.7)	2 (16.7)	
11–15/day	0 (0)	8 (40)	7 (35)	5 (25)	
16–20/day	0 (0)	3 (25)	4 (33.3)	5 (41.7)	
Duration					
< 5 years	0 (0)	3 (60)	1 (20)	1 (20)	0.84**
5–10 years	0 (0)	4 (36.4)	3 (27.3)	4 (36.4)	
11–15 years	0 (0)	6 (26.1)	10 (43.5)	7 (30.4)	
16–20 years	0 (0)	7 (33.3)	6 (28.6)	8 (38.1)	
Site					
Buccal mucosa	0 (0)	11 (35.5)	9 (29)	11 (35.5)	0.82**
Tongue	0 (0)	3 (30)	4 (40)	3 (30)	
Lower buccal sulcus	0 (0)	2 (50)	2 (50)	0 (0)	
Lower lip	0 (0)	2 (66.7)	0 (0)	1 (33.3)	
Palate	0 (0)	1 (25)	2 (50)	1 (25)	
Retromolar area	0 (0)	1 (20)	1 (20)	3 (60)	
Upper sulcus	0 (0)	0 (0)	2 (66.7)	1 (33.3)	

^^^Values represented as Median (Interquartile Range)

*Chi-Square

**Fisher Exact test

^Ò^Kruskal Wallis Test

The median (IQR) of salivary protein levels observed in three study groups of OSCC and in control group were as follows: well differentiated OSCC 15.11 (13.88), moderately differentiated OSCC 26.5 (29.16), poorly differentiated OSCC 11.38 (9.81) mg/ml and in control group 3.2 (3.03) as shown in Table 3. Statistically significant difference with *p* value (< 0.001) was observed in salivary protein levels between OSCC and control group. Highest protein levels were observed in moderately differentiated OSCC followed by well differentiated OSCC and poorly differentiated OSCC. Lowest salivary protein levels were observed in control group. In pairwise comparison between three groups of OSCC with control group, significance was observed in salivary total protein levels between well differentiated and control group, moderately differentiated and control group and poorly differentiated and control group.

**Table 3:** Salivary total protein levels in OSCC and control groups

	Median (IQR)	*p* value^ò^
Groups		
Healthy controls (N = 20)	3.2 (3.03)	
Well differentiated OSCC (N = 20)	15.11 (13.88)	
Moderately differentiated OSCC (N = 20)	26.5 (29.16)	< 0.001
Poorly differentiated OSCC (N = 20)	11.38 (9.81)	
Comparison of OSCC vs Healthy controls		*p* value^ø^
Well differentiated OSCC		**< **0.001
Moderately differentiated OSCC		< 0.001
Poorly differentiated OSCC		0.005

^Ò^Kruskal Wallis test

^ø^Post hoc test (Dunnett’s test)

Among total 60 OSCC patients, Cathepsin B in saliva was detected in 45 (70%) cases where as in the control group salivary Cathepsin B was detected in only 3 (15%) cases. The median (IQR) of salivary Cathepsin B levels observed in three groups of OSCC were as follows: well differentiated OSCC 3.57 (2.85) ng/ml, moderately differentiated OSCC 0.7 (1.54) ng/ml and poorly differentiated OSCC patients 1.76 (6.6) ng/ml. Statistically significant difference with *p* value (< 0.001) was observed in salivary Cathepsin B levels between OSCC and control group. Highest salivary Cathepsin B levels were observed in well differentiated OSCC followed by poorly differentiated OSCC and moderately differentiated OSCC as shown in Table 4. In pairwise comparison between three groups of OSCC with control group, highly significant *p* value was observed in salivary Cathepsin B levels between well differentiated and control group and poorly differentiated and control group.

**Table 4:** Salivary cathepsin B levels in OSCC and control group

	Median (IQR)	*p* value^ò^
Groups		
Healthy controls	0.0 (0.0)	
Well differentiated OSCC	3.57 (2.85)	< 0.001
Moderately differentiated OSCC	0.7 (1.54)	
Poorly differentiated OSCC	1.76 (6.6)	
Tumor size		< 0.001
Healthy controls	0.0 (0.0)	
T1	0.87 (0.68)	
T2	1.46 (1.03)	
T3	1.89 (3.3)	
T4	3.95 (5.82)	
Comparison of OSCC vs Healthy controls		*p* value^ø^
Well differentiated OSCC		< 0.001
Moderately differentiated OSCC		0.65
Poorly differentiated OSCC		< 0.001

^**Ò**^Kruskal Wallis test

^**ø**^Post Hoc test (Dunnett’s test)

Among the 60 patients of OSCC according to tumor size, T1 comprised 5 (6.3%), T2 comprised 9 (11.3%), T3 comprised 17 (21.3%) and T4 comprised 29 (36.3%) OSCC cases respectively. Cathepsin B levels were also significantly increased with *p* value (< 0.001) according to tumor size as compared to healthy controls as shown in Table 4.

No significant association of salivary Cathepsin B levels with age, gender, site, frequency and duration of tobacco intake were observed as shown in Table 5.

**Table 5:** Association of Cathepsin B with patient related factors

Category	N = 80%	Mean Cathepsin B level ± SD	*p* value
Gender			
Male	55 (68.7)	2.1 ± 3.0	0.95**
Female	25 (31.3)	2.1 ± 2.3	
Frequency of tobacco intake^Ϯ^			
1–5/day	16 (26.7)	2.4 ± 2.8	0.09*
6–10/day	13 (21.7)	3.1 ± 3.1	
11–15/day	19 (31.7)	3.5 ± 2.5	
16–20/day	12 (20.0)	1.2 ± 0.8	
Duration of tobacco intake^Ϯ^			
< 5 years	5 (8.3)	2.3 ± 2.7	0.91*
5–10 years	11 (18.3)	2.8 ± 2.6	
11–15 years	23 (38.3)	2.4 ± 2.6	
16–20 years	21 (35.0)	2.9 ± 2.8	
Site of lesion^Ϯ^			
Buccal mucosa	31 (51.7)	2.6 ± 2.4	0.77*
Tongue	10 (16.7)	2.0 ± 2.9	
Lower buccal sulcus	4 (6.7)	2.4 ± 1.4	
Lower lip	3 (5.0)	2.7 ± 2.4	
Palate	4 (6.7)	1.9 ± 2.1	
Retromolar area	5 (8.3)	4.2 ± 3.5	
Upper sulcus	3 (5.0)	3.9 ± 5.1	
Age^^^ 0.083 (0.465)			0.465^

^Ϯ^n = 60 i.e., only for OSCC patients

^Pearson Correlation

*One way ANOVA

**T-test

Univariate and multivariate analyses was performed to observe association of salivary Cathepsin B levels with clinicopathological characteristics and study groups. The only variable which observed significant association with Cathepsin B levels was the OSCC/non-OSCC group. Therefore, execution of multivariate analysis was not possible. Interpretations of regression (B) coefficient showed estimated increase in salivary Cathepsin B levels for well differentiated OSCC patients 3.37 ng/ml, for moderately differentiated OSCC patients 0.63 ng/ml and for poorly differentiated OSCC patients 2.99 ng/ml were observed respectively as shown in Table 6.

**Table 6:** Analysis of the association of estimated mean Cathepsin B levels with independent variables by linear regression

Independent variables	β (standard error)	*p* value*
Groups		
Well differentiated OSCC	3.37 (0.66)	< 0.001
Moderately differentiated OSCC	0.63 (0.66)	0.34
Poorly differentiated	2.99 (0.66)	< 0.001
Healthy controls	Ref	
Proteins(mg/ml)	0.009 (0.02)	0.61
Gender		
Male	0.04 (0.61)	0.95
Female	Ref	

*Linear Regression

**Fig. 1:**
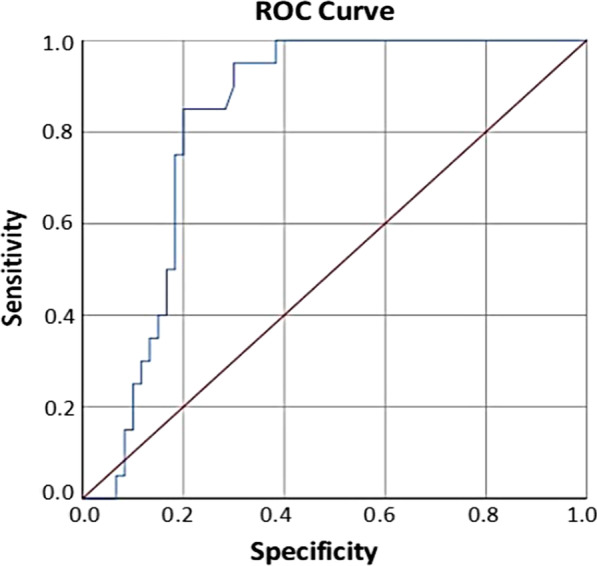
Receiver operating characteristics (ROC) curve analysis of the predictive value of Cathepsin B for OSCC

By using Receiving Operative Curve (ROC), sensitivity 85% and specificity 80% with highly significant *p* value < 0.001 for Cathepsin B was observed as shown in Table 7 and Fig. 1.

**Table 7:** Cathepsin B cut-off for OSCC using receiving operative curve (ROC)

Group	Cut-off	Sensitivity	Specificity	AUC (*p* value)
OSCC	≥ 2.0	85%	80%	83% (< 0.001)

AUC, area under the curve

## Discussion

Oral squamous cell carcinoma worldwide is one of the most common malignant tumor, which has high rates of morbidity and mortality [27]. The main reason is that most OSCC cases when diagnosed are in advanced stages [28]. The diagnosis and monitoring of OSCC by using salivary biomarkers is considered a promising approach for lowering oral cancer burden and increase the overall survival rate [29]. This is the first study in which we have analyzed salivary levels of Cathepsin B in different histological grades of OSCC.

Cathepsin B role in cancer is attributed to extracellular matrix degradation resulting in tumor invasion and metastasis [23]. It is considered a promising biomarker as increased expression of Cathepsin B was reported in various types of metastasis and invasive cancers such as lung, breast, ovary, colon, gastric, nasopharyngeal and oral cancer [23]. Xu et al*.* in his research has reported increased expression of Cathepsin B in gastric carcinoma as compared to normal tissue which is found to be significantly associated with tumor size (*p* < 0.001), Tumor Node Metastasis (TNM) stage (*p* < 0.001) and decreased overall survival (*p* < 0.001) [30]. In malignant ovarian cancers, Cathepsin B levels were significantly elevated in cystic fluid as reported by Kolwijck et al. [31]. Chan et al. in his research analyzed Cathepsin B in 588 colon cancer patients and found that 82% patients were positive for Cathepsin B, which was further confirmed by Cavallo-Medved et al*.* and he concluded that elevated levels of Cathepsin B were associated with poor prognosis [32]. In nasopharyngeal carcinoma, Cathepsin B levels were significantly elevated in serum as compared to healthy controls which was significantly associated with TNM (*p* = 0.001) as observed by Tan et al. [24]. In breast cancer patients, Schraufstatter et al*.* observed increased Cathepsin B in serum with *p* value (0.015) as compared to normal control [31].

In our study, we observed prevalence of OSCC is higher in males as compared to females. Buccal mucosa as the most common site of OSCC occurrence was observed in our study. Tobacco consumption of both forms smoking and smokeless tobacco for greater than 5 years duration caused increase in no. of OSCC cases.

In our study, salivary total protein levels were increased significantly with *p* value (< 0.001) in patients of OSCC as compared to control group. Shivashankara along with Kavya Prabhu and Mussavira et al*.* has also reported in their studies that salivary protein levels were increased significantly in OSCC patients in comparison to control group [33, 34]. Proteins are accountable for most functions of saliva such as physical protection, lubrication, buffering, tooth integrity and antibacterial activity [35]. Total salivary proteins were increased in patients of OSCC probably due to the ongoing inflammatory response. It triggers the sympathetic activity which increases the synthesis and production of some proteins to increase the shielding effect of saliva and provide protective function to combat against OSCC [35]. Furthermore, increase in salivary total proteins occurs to combat the violation and aberration in capillaries and mucosal lining as a result of inflammation in OSCC [36]. In our study, we observed highest levels of total salivary protein in moderately differentiated followed by well differentiated and poorly differentiated OSCC. This could be due to the fact that total salivary protein levels in patients of OSCC is dependent on several factors relatable to patients such as (diet, gender and age) and factors related to disease such as (infection, metastasis and lymph node invasion) [37].

In our study, the levels of salivary Cathepsin B were determined in OSCC patients in different histological grades and according to tumor size. Cathepsin B was observed in 45 (70%) patients out of total 60 OSCC patients and in control group Cathepsin B was detected in only 3 (15%) of healthy controls. It was observed that salivary levels of Cathepsin B were increased significantly with *p* value (< 0.001) in patients of OSCC according to both histological grades and tumor size as compared to control group. Increased expression of Cathepsin B in tissues, serum and cell line were reported by several researches. Yang et al. in his research has observed increased CTSB expression in patients of OSCC, which was associated with higher tumor grade (*p* = 0.008) and lymph node metastasis (*p* = 0.007) [25]. Yang et al*.* has reported increased CTSB protein and mRNA levels in cell lines and tissues of OSCC patients as compared to adjacent non-malignant tissues by polymerase chain reaction (PCR), western blotting and immunohistochemistry analysis [38]. Saleh et al*.* in tongue cancer patients has observed elevated Cathepsin B in serum and tumor tissues associated with high tumor grade [39].

The highest salivary Cathepsin B levels in well differentiated group followed by poorly differentiated group, moderately differentiated OSCC group and controls were observed in our study. The variable results observed in our study, could be due to the fact that Broder’s histopathological grading of OSCC provides us information only about degree of differentiation of cell which is only one parameter [40]. However, prognosis of OSCC apart from histological grading also depends on other factors such as tumor size, lymph node invasion and metastasis [41]. Furthermore, as Cathepsin B is an inflammatory marker and more inflammatory cells are present in initial grades of OSCC that’s why this may be the plausible reason we observed increased Cathepsin B salivary levels in well differentiated OSCC [42]. Kullage et al. in his research had also reported high inflammatory cell count in patients of well differentiated OSCC in correspondence to moderate and poorly differentiated OSCC [43]. Cathepsin B role in cancer is related to basement membrane and extracellular matrix protein degradation, a mechanism which is responsible for metastasis and tumor invasion of cancer cells. It activates and initiates a proteolytic cascade in which urokinase plasminogen activator, matrix metalloproteinase and plasminogen all together causes degradation of extracellular matrix components. Cathepsin B also causes degradation of cell adhesion protein E cadherin at adhering junctions which causes detachment of cells. This altogether results in cell invasion, progression of tumor and metastases [44, 45]. That might be the plausible reason, we observed increased salivary Cathepsin B levels in poorly differentiated OSCC.

## Conclusion

In our study, salivary Cathepsin B levels were significantly increased in patients of OSCC as compared to healthy controls. Thus, it can be considered a helpful and beneficial salivary biomarker for diagnosis and monitoring of OSCC in different histological grades. Timely detection and treatment will increase the survival rate of OSCC and further reduce the mortality and morbidity rate. The results of the present study made the way for future studies on Cathepsin B as a non-invasive tool salivary biomarker for diagnosis and monitoring of OSCC.

### Limitations of the study

There are some limitations, which are present in our study. The sample size is small and this study contains research participants only from one tertiary care hospital of Karachi. We have only analyzed cathepsin B levels in patients of OSCC, patients with benign lesions and/or other chronic inflammatory oral diseases were not included in our study. Furthermore, we have analyzed OSCC samples only on the basis of histopathological grading and tumor size. Information about node status, distant metastasis, tumor staging and cancer specific survival was not recorded in our study. Further, research validation with large sample size and multicenter study, which represent the entire population of Pakistan with considerations of all parameters of OSCC, should be conducted in future.

## Data Availability

The raw data is a property of Dow University of Health Sciences. The data set used and analyzed during the current study are available from corresponding author on reasonable request.

## Abbreviations

OSCC: Oral squamous cell carcinoma
CTSB: Cathepsin B
ELISA: Enzyme linked immunosorbent assay
TNM: Tumor node metastasis
PCR: Polymerase chain reaction
ROC: Receiver operating characteristics
